# Anti-inflammatory and protective effects of *Pimpinella candolleana* on ulcerative colitis in rats: a comprehensive study of quality, chemical composition, and molecular mechanisms

**DOI:** 10.3389/fphar.2024.1328977

**Published:** 2024-04-05

**Authors:** Xiaoqi Liu, Hai Xiao, Mingxia Luo, Junpeng Meng, Lin Zhong, Tao Wu, Yongxia Zhao, Faming Wu, Jian Xie

**Affiliations:** ^1^ Department of Pharmacognosy, Zunyi Medical University, Zunyi, China; ^2^ Maternal and Child Health Carelhospita, Zunyi, China; ^3^ Department of Medical Genetics, Zunyi Medical University, Zunyi, China; ^4^ Guizhou Medical and Health Industry Research Institute, Zunyi Medical University, Zunyi, China

**Keywords:** *Pimpinella candolleana* Wight et Arn., ulcerative colitis, quality evaluation, anti-inflammatory, molecular mechanism

## Abstract

**Introduction:** *P. candolleana* Wight et Arn. Is a traditional Chinese herbal medicine used by the Gelao nationality in southwest China, has been historically applied to treat various gastrointestinal disorders. Despite its traditional usage, scientific evidence elucidating its efficacy and mechanisms in treating ulcerative colitis (UC) remains sparse. This study aimed to determine the quality and chemical composition of *Pimpinella candolleana* and to identify its potential therapeutic targets and mechanisms in acetic acid-induced ulcerative colitis (UC) rats through integrated approaches.

**Methods:** Morphological and microscopic characteristics, thin layer chromatography (TLC) identification, and quantitative analysis of *P. candolleana* were performed. UPLC-Q-TOF-MS, network pharmacology, and molecular docking were used to identify its chemical composition and predict its related targets in UC. Furthermore, a rat model was established to evaluate the therapeutic effect and potential mechanism of *P. candolleana* on UC.

**Results:** Microscopic identification revealed irregular and radial arrangement of the xylem in *P. candolleana*, with a light green cross-section and large medullary cells. UPLC-Q-TOF-MS analysis detected and analyzed 570 metabolites, including flavonoids, coumarins, and terpenoids. Network pharmacology identified 12 effective components and 176 target genes, with 96 common targets for *P. candolleana*-UC, including quercetin, luteolin, and nobiletin as key anti-inflammatory components. GO and KEGG revealed the potential involvement of their targets in RELA, JUN, TNF, IKBKB, PTGS2, and CHUK, with action pathways such as PI3K-Akt, TNF, IL-17, and apoptosis. Molecular docking demonstrated strong affinity and binding between these key components (quercetin, luteolin, and nobiletin) and the key targets of the pathway, including JUN and TNF. Treatment with *P. candolleana* improved body weight loss, the disease activity index, and colonic histological damage in UC rats. *Pimpinella candolleana* also modulated the levels of IL-2 and IL-6 in UC rats, reduced the expression of pro-inflammatory cytokines such as IL-6, MAPK8, TNF-α, CHUK, and IKBKB mRNA, and decreased the expression of TNF, IKBKB, JUN, and CHUK proteins in the colon of UC rats, thereby reducing inflammation and alleviating UC symptoms.

**Conclusion:** *P. candolleana* exerts its protective effect on UC by reducing the expression of proinflammatory cytokines and inhibiting inflammation, providing scientific evidence for its traditional use in treating gastrointestinal diseases. This study highlights the potential of *P. candolleana* as a natural therapeutic agent for UC and contributes to the development of novel medicines for UC treatment.

## TIntroduction

*Pimpinella candolleana* Wight et Arn., widely recognized and utilized by the Gelao ethnic group in Southwest China, is a traditional herbal medicine with a rich historical background, as documented in “Sichuan Traditional Chinese Medicine Annals” (Collaborative writing group Sichuan Journal of Traditional Chinese Medicine, 1980), “DianNan herbal” (Lan, 2004), and “Yunnan herbal medicine” (Revolutionary Committee of Yunnan Provincial Health Bureau, 1971). Renowned for its extensive medicinal properties, including promoting “Qi”, alleviating pain, warming the spleen and stomach, dispelling cold, relieving rheumatism, stimulating blood flow, and aiding in detoxification and detumescence, P. candolleana is primarily used for treating gastrointestinal issues such as abdominal pain and diarrhea and is favored by local communities and widely incorporated in township hospitals in Guizhou and Yunnan. Despite its widespread use, insufficient research has been conducted on the evaluation of its quality, identification of its active compounds, and exploration of its pharmacological actions and efficacy (Wei and Gui, 1992). Therefore, it is essential to conduct comprehensive quality assessments and pharmacodynamic studies on *Pimpinella candolleana*, with the ultimate goal of safeguarding, developing, and optimally utilizing this valuable traditional Chinese medicine (Liang et al., 2003; Liu et al., 2021).

Ulcerative colitis (UC), a chronic inflammatory bowel disease affecting the colon and rectum, is characterized by symptoms such as inflammation, ulceration, and bleeding of the intestinal mucosa. The etiology of UC is believed to be multifactorial, involving genetic predispositions and environmental influences (Wang et al., 2019; Yousefi-Manesh et al., 2020; Motavallian et al., 2021). The escalating global incidence of UC not only impairs human health (Ghasemi-Pirbaluti et al., 2017) but also raises the risk of colon cancer. Conventional therapeutic regimens, encompassing aminosalicylates, corticosteroids, immunosuppressants, and biological agents, have been employed in UC management. However, the suboptimal efficacy and pronounced side effects of these drugs are undeniable (Zhao et al., 2022). The urgency to discover safer and more effective alternative UC therapies cannot be overemphasized. Chinese herbal medicines, known for their ability to modulate the inflammatory response with minimal side effects, present a promising avenue. Among these, *P. candolleana*, leveraged by the Gelao for its traditional uses, emerges as a candidate (Xing et al., 2011). Preliminary investigations suggest its bioactive constituents, such as flavonoids, coumarins, and terpenoids, may offer anti-inflammatory and antioxidant benefits. However, a detailed understanding of its chemical profile and the molecular mechanisms by which it influences UC is yet to be established (Wei et al., 2005; Zhao et al., 2007).

**FIGURE 1:**
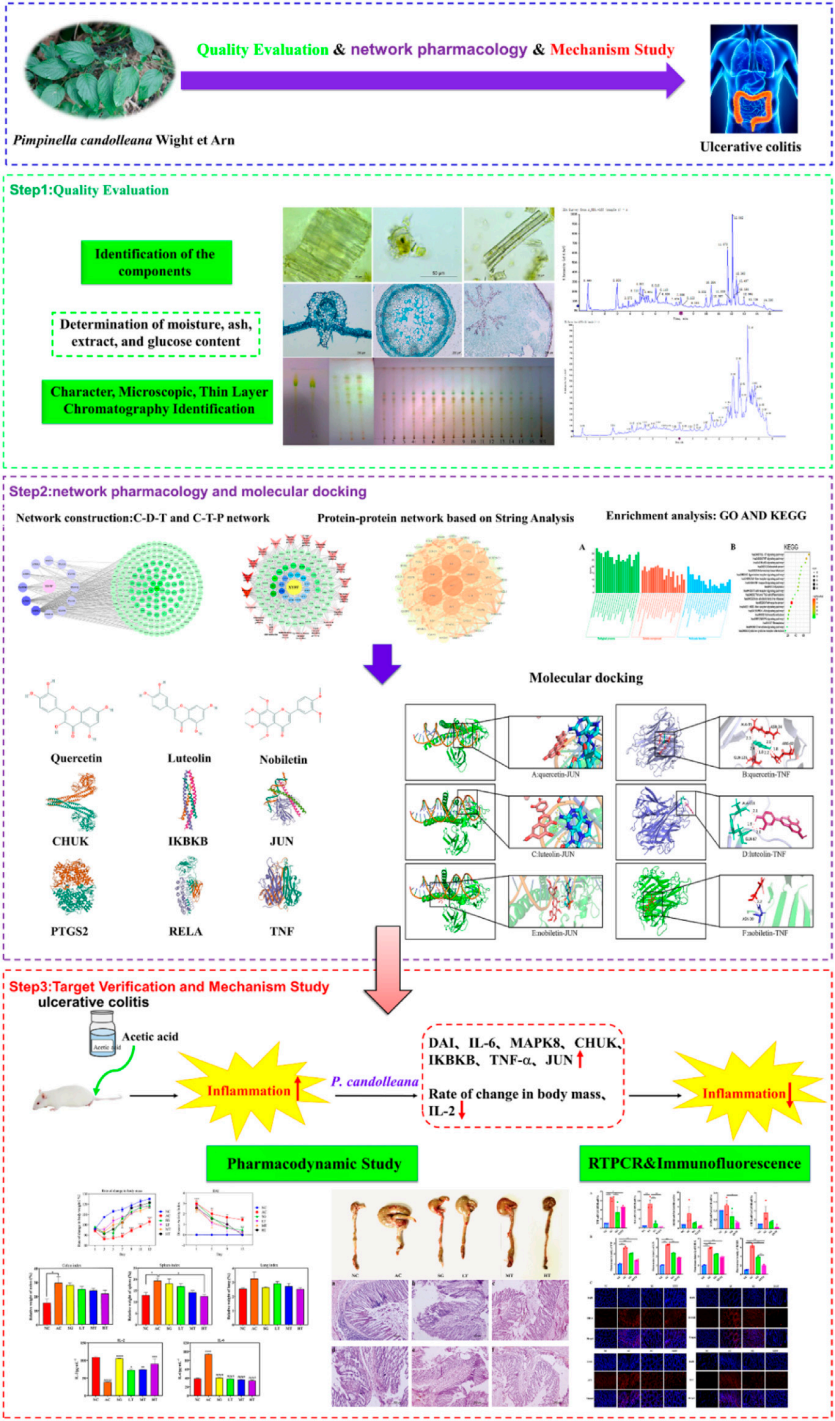
Technical strategy of the current study.

In this study, we endeavored to elucidate the anti-inflammatory mechanism and therapeutic impact of *P. candolleana* on UC by employing an integrated approach encompassing quality evaluation, network pharmacology, UPLC-Q-TOF-MS, molecular docking and *in vivo* experiments (Zhao et al., 2022; He et al., 2022; Wang et al., 2022). To establish preliminary quality evaluation standards for *P. candolleana*, we conducted a comprehensive assessment of its macroscopic and microscopic features, thin layer chromatography, moisture content, ash content, extractability, and total polysaccharide content, as outlined in the “Pharmacopoeia of the People’s Republic of China” (2020 edition). Additionally, through network pharmacology, we identified the key components and targets of *P. candolleana* in UC, revealing potential pathways and mechanisms underlying its anti-inflammatory effect (Yang et al., 2023). The chemical composition of the *P. candolleana* extract was analyzed using UPLC-Q-TOF-MS, and molecular docking was performed to verify the binding affinity of the key components with the identified targets. By integrating these methods, we aimed to provide a comprehensive and systematic understanding of the anti-inflammatory mechanism and therapeutic effect of *P. candolleana* on UC. Our study also involved *in vivo* experiments using an acetic acid-induced UC rat model to evaluate the therapeutic effect of *P. candolleana* extract and investigate its influence on the expression of proinflammatory cytokines and related proteins in colon tissue. Please refer to Figure 1 for an overview of our experimental technical strategy.

## Materials and methods

### Reagent

Sulfasalazine enteric-coated tablets (H31020557) were purchased from Shanghai Xinyi Tianping Pharmaceutical Co., Ltd. Glacial acetic acid (CAS No. 64-19-7) was purchased from Chengdu Kelong Chemical Reagent Factory, and anhydrous ethanol (analytical grade, batch No.202012) was purchased from Chengdu Jinshan Chemical Reagent Co., Ltd. Acetonitrile (Merck, 1499230-935), ammonium acetate (Sigma, 70221), and D-(+)-glucose (>99.5% purity) were purchased from Shenggong Bioengineering (Shanghai) Co., Ltd. 4% tissue cell fixative (No.20210626), hematoxylin and eosin (HE) staining kit (No.20211016), rat interleukin-2 ELISA kit (batch No.20210804), rat interleukin-6 ELISA kit (No.20210728), Reverse transcription reaction reagent (RR037A) and TB Green Premix Ex TaqII (RR820A) were purchased from Takara Biomedical Technology (Beijing) Co., Ltd. DAPI staining reagent (G1012), anti-fluorescence quenching sealing tablets (G1401), JUN (GB11071), TNF-α (GB11188), CHUK (GB11923), immunofluorescence secondary antibody-GY3 labeled goat anti-rabbit IgG (GB21303), bovine serum albumin BSA (GC305010), citric acid antigen repair solution (G1202), and tissue autofluorescence quenching agent (G1221) were all purchased from Wuhan Servicebio Technology Co., Ltd. The primer synthesis (231216282) was purchased from Beijing Lusen Biotechnology Co., Ltd. Anti-IKK beta antibody (EPR6043) were purchased from Aibokang (Shanghai) Trading Co., Ltd.

### Quality evaluation of *Pimpinella candolleana*

#### Preparation of medicinal materials

Fresh plants were collected in Simian Village, Huangdou Town, Wuchuan County, Zunyi City (107 38′45″E, 28 19′54″N, 1000 m above sea level) and were identified as *P. candolleana* Wight et Arn. (Umbelliferae) by Dr. Wu (School of Pharmacy, Zunyi Medical University.). Then, impurities were removed from fresh *P. candolleana*, washed, drained, dried at 50°C, pulverized, numbered, and stored in a refrigerator at −4°C to obtain *P. candolleana* medicinal material.

#### Traits, TLC, water content, total ash and exudates

The plant height, taproot length, diameter, dry weight, color, odor, texture, and cross-section of fresh were measured and described according to the description method of medicinal materials in the Pharmacopoeia of the People’s Republic of China (2020 edition, Vol. 1). According to the Pharmacopoeia of the People’s Republic of China (2020 edition, Vol. 5), the root and stem cross sections, upper and lower surfaces of leaves and medicinal powder of fresh *P. candolleana* were identified microscopically. *Pimpinella candolleana* was identified by TLC (NO. 0502). The water content (NO. 0832), total ash content (NO. 2302) and extract (NO. 2201) of medicinal materials were checked.

#### Determination of total polysaccharide content

We prepared a series of glucose standard solutions with different concentrations by dissolving 0.0102 g of glucose in 100 mL of deionized water, transferring 0.1, 0.2, 0.3, 0.4 and 0.5 mL of the solution into dry test tubes, adding deionized water to make up to 1 mL, adding 0.4 mL of 5% phenol solution, mixing well, adding 2 mL of concentrated sulfuric acid, shaking well, and cooling. We extracted the polysaccharides from *P. candolleana* medicinal powder by using the ethanol precipitation method as follows: we refluxed 1 g of the powder with 100 mL of 80% ethanol for 4 h in a Soxhlet extractor, evaporated the ethanol, added 100 mL of anhydrous ethanol, refluxed for another 1 h, and centrifuged; we collected 2 mL of the supernatant, added 10 mL of anhydrous ethanol, and left it overnight; after centrifugation, we washed the precipitate twice with 80% ethanol, dissolved it in 10 mL of deionized water, and measured 1 mL of the solution; we added 400 μL of 5% phenol, mixed well, added 2 mL of concentrated sulfuric acid, mixed well, and cooled to determine the absorbance at a wavelength of 488 nm.

### UPLC-Q-TOF-MS was used to detect the composition of *Pimpinella candolleana*

#### Sample extraction method

*Pimpinella candolleana* powder was slowly thawed at 4°C, and a proper amount of sample was collected and mixed with a precooled methanol/acetonitrile/water solution (2:2:1, v/v) by using a vortex. The mixture was sonicated at low temperature for 30 min, frozen at −20°C for 10 min and centrifuged at 14000 *g* at 4°C for 20 min. The supernatant was vacuum-dried and reconstituted in an acetonitrile water solution (acetonitrile:water = 1:1, v/v) for mass spectrometric analysis.

#### UPLC-Q-TOF-MS conditions

The analysis was performed on an Agilent 1290 Infinity LC Ultra Performance Liquid Chromatography (UHPLC) system coupled with an Agilent 6545 Q-TOF mass spectrometer. The separation was carried out on an Agilent C-18 column (100 mm × 2.1 mm, 1.8 μm); the column temperature was 40°C; the flow rate was 0.4 mL/min; the injection volume was 2 μL; the mobile phase consisted of A: water +25 mM ammonium acetate +0.5% formic acid, and B: methanol; the gradient elution program was as follows: 0–0.5 min, 5% B; 0.5–10 min, B changed linearly from 5% to 100%; 10.0–12.0 min, B maintained at 100%; 12.0–12.1 min, B changed linearly from 100% to 5%; 12.1–16 min, B maintained at 5%. The samples were kept in a 4°C autosampler throughout the analysis. The ESI source conditions after HILIC chromatographic separation were as follows: ion source gas 1 (GaS 1): 60, ion source gas 2 (GaS 2): 60, curtain gas (Cur): 30, source temperature: 600°C, ion spray voltage floating (ISDF) 5500 V (positive and negative modes); TOF MS scan m/z range: 60–1000 Da, product ion scan m/z range: 25–1000 Da, TOF MS scan accumulation time 0.20 s/spectra, product ion scan accumulation time 0.05 s/spectra. The tandem mass spectrum was obtained using information-dependent acquisition (IDA) in high-sensitivity mode, declustering potential (DP): 60 V (both positive and negative modes), and collaboration per cycle: 10.

### Network pharmacology-based analysis

#### Screening of action targets of *Pimpinella candolleana*

Since there is little research on *P. candolleana* and its components are not fully analyzed, we searched the TCMSP database (https://old.tcmsp-e.com/tcmsp.php) with the INCHIKEY number of the 570 components detected by UPLC-Q-TOF-MS. We utilized PubChem (https://pubchem.ncbi.nlm.nih.gov/) and UNIPROT (https://www.uniprot.org/) to obtain the related targets of the active components and used Cytoscape 3.7.0 (https://cytoscape.org/) to draw the active component-gene target network.

#### Collection of gene targets for UC

The key word “Colitis” was searched in GeneCards (https://www.genecards.org/), and 5492 genes related to colitis were exported to Excel. With a relevance score ≥1.1715 as the criterion, 1199 important target genes of colitis were selected. After removing duplicates, we used Venny software (https://bioinfogp.cnb.csic.es/tools/venny/y/) to visualize the overlap between the drug target genes and the disease target genes and obtained their common target genes, which are potential targets for the drug treatment of diseases.

#### Network construction and enrichment analysis

The potential active component-target network of *P. candolleana* was constructed and analyzed to illustrate the crucial targets via Cytoscape v3.7.2. Then, the protein‒protein interactions (PPIs) of core targets were derived from the STRING 11.0 database (https://string-db.org/). Then, the biological functions of the core targets were determined through Gene Ontology (GO) enrichment annotation and Kyoto Encyclopedia of Genes and Genomes (KEGG) pathway analysis in the Metascape database, with *p* < 0.01.

#### Molecular docking verification

Molecular docking was used to simulate the potential interactions between components of *P. candolleana* and the core targets. The structure files of components and target proteins were downloaded from the TCMSP database and PDB database, respectively. After removing water molecules and ligands, the target protein interacted with components in the binding site via Autodock v 1.4.0.

### Anti-UC of *Pimpinella candolleana*

#### Construction of the UC rat model

Animal experiments were conducted in full compliance with animal ethical requirements and approved by the Animal Ethics Review Committee of Zunyi Medical University (ZMU21-2203-433).

Forty-eight SPF male SD rats weighing 280–320 g (License No.: SCXK (Xiang) 2019-0004) were randomly divided into a normal group, model group, positive drug group, and low-, medium- and high-dose groups of *P. candolleana* (*n* = 8). After adaptive feeding for 1 week, the rats were deprived of food for 24 h and anesthetized with pentobarbital sodium. A catheter with an outer diameter of 2 mm and a length of 8 cm was inserted reversely from the anus of rats into the colon 8 cm away from the anus. The normal group was enema with 2 mL of physiological saline, and the others were slowly injected with 2 mL of 3% acetic acid into the colon. After 10 s, 2 mL of physiological saline was injected immediately to wash the colon, and the rats were put back into the cage after waking up. Diet and water were given after 4 h. At 9:00 a.m. every day, rats in the model group were intragastrically administered physiological saline, while rats in the positive medication group were intragastrically administered 5 mg/kg sulfasalazine, and low, middle and high doses of *P. candolleana* were given 600 mg/kg, 1200 mg/kg and 1800 mg/kg, respectively, for 14 days. Acetic acid-induced colitis is a commonly used animal model that closely mimics human UC(Gao et al., 2019). After *P. candolleana* intervention, there were no deaths in the normal group (NC), while the mortality rate in the positive control group (SG) was 62.5%. The mortality rates of the low-, middle-, and high-dose groups (LT, MT, HT) were 12.5%, 37.5%, and 12.5%, respectively. The average mortality rate in the *P. candolleana* treatment group was 20.83%. These results indicate that *P. candolleana* has a positive effect on reducing the mortality rate of UC. Therefore, we conducted *in vivo* experiments using acetic acid-induced UC rats.

#### Sampling and processing

The changes in fur, mental state and stool viscosity of rats in each group were observed, and the DAI score was calculated. DAI= (body weight decline rate + stool traits score + stool occult blood score)/3. Twelve hours after drug administration, the rats were deprived of food and water, and blood was collected from the eyes after anesthesia. Serum was separated and stored at −20°C. After the rats were sacrificed, the spleen, kidney and liver tissues were weighed, and the organ index (organ index% = organ weight/body mass ×100%) was calculated according to the formula. The abdomen was cut open, the colon was removed, the intestinal contents were washed with physiological saline and then dried and weighed, and injury to the colonic mucosa was observed by taking photos. The remaining tissues were sealed in 4% paraformaldehyde solution and stored at 4°C in the dark.

#### Histopathological observation and detection of inflammatory factors

Colon tissue fixed with 4% formaldehyde solution was routinely embedded in paraffin, sectioned, stained with hematoxylin-eosin dye, dehydrated and sealed, and pathological changes, such as structural integrity, congestion, ulcer and lymphocyte infiltration, were observed under a microscope. The contents of IL-2 and IL-6 in the serum of rats were detected according to the instructions of ELISA kits.

#### Real-time PCR

The transcription levels of the JUN, TNF-α, CHUK, IKBKB, and IL-6 genes in rat colon tissues were examined using reverse transcription quantitative polymerase chain reaction (RT‒qPCR). Total RNA from the colon tissues was extracted using TRIzol reagent, chloroform, and isopropanol, and RNA quality was assessed with a Nanodrop 2000 Spectrophotometer. The extracted RNA was reverse-transcribed using a two-step reverse transcription kit under the following conditions: 37°C for 15 min, 85°C for 5 s, and 4°C indefinitely. The resulting complementary DNA (cDNA) was stored at −80°C.

RT‒qPCR was performed using TB Green Premix Ex Taq II reagent with gene-specific primers, and the relative mRNA levels of the target genes were quantified using the 2^−ΔΔCT^ method, with glyceraldehyde 3-phosphate dehydrogenase (GAPDH) serving as an internal control. The PCR cycling conditions were set as follows: 95°C for 30 s, 95°C for 5 s, 60°C for 30 s, followed by 39 cycles, 95°C for 10 s, and a melt curve from 65.0°C to 95.0°C with a 0.5°C increment for 5 s plus plate read.

The primer sequences for the IKBKB gene (5′–3′) were forward: GAT​CTG​TCT​TGG​CGC​CTC​TT, and reverse: TTT​AGC​AGC​TCA​CAG​GTC​CC. For the IL-6 gene, the primer sequences (5′–3′) were forward: GCC​CTT​CAG​GAA​CAG​CTA​TG, and reverse: CAG​AAT​TGC​CAT​TGC​ACA​AC. The TNF-α gene primer sequences (5′–3′) were forward: TGA​TCG​GTC​CCA​ACA​AGG​A, and reverse: CGC​TTG​GTG​GTT​TGC​TAC​GA. The MAPK8 gene primer sequences (5′–3′) were forward: ACA​CCA​CAG​AAA​TCC​CTA​GAA​G, and reverse: CAC​AGC​ATT​TGA​TAG​AGA​AGG​T. Last, for the CHUK gene, the primer sequences (5′–3′) were forward: AAA​GAC​CAG​GGT​GGG​TGA​AG, and reverse: GAC​TGC​CGT​TGC​AAT​GGT​TAC.

#### Immunofluorescence staining

Paraffin-embedded colon tissue sections from rats were deparaffinized in water and subjected to heat-induced antigen retrieval using citrate buffer (pH 6.0), followed by natural cooling. The slides were washed with PBS and blocked with 3% BSA for 30 min, followed by incubation with primary antibodies at 4°C overnight. The next day, the slides were washed with PBS and incubated with secondary antibodies in the dark at room temperature for 50 min. The nuclei were stained with DAPI, and the slides were washed with PBS and mounted with anti-fade mounting medium. The slides were observed, and images were captured using a Nikon upright fluorescence microscope. The fluorescence intensity of each group was analyzed using ImageJ software, and the relative expression levels were calculated.

### Statistical processing

SPSS 18.0 and GraphPad Prism 9 were used for analysis. The experimental data are expressed as x ± s, and one-way ANOVA was used for comparisons among groups. *p* < 0.05 indicates that the difference is statistically significant.

## Results

### Characterization and quality evaluation of *Pimpinella candolleana*

#### Morphological and microscopic features

The morphological and microscopic features of *P. candolleana* plants collected from different locations in Guizhou Province, China, were observed. These plants were identified as perennial herbs, with a total length of 31.20–81.62 cm, a dry weight of 0.2116–0.4736 g, and a ratio of dry weight of the underground part to that of the aboveground part of 0.1716–0.3306. The roots were fine and long, with a length of 0.49–4.03 cm and a diameter of 0.29–0.43 cm. The roots were pale yellow, shrunken, brittle and easily broken, and the cross section was white. The stems were erect, dark green on the surface, slightly hard, breakable, and light green in cross section. The leaves were heart-shaped, with serrated edges. The plant material emitted a mild odor and possessed a slightly bitter taste. Significantly, notable variations were observed in the total length, dry weight, and ratio of the dry weight of the underground part to that of the aboveground part among different batches of *P. candolleana* (Table 1).

**TABLE 1:** Character identification of *Pimpinella candolleana*.

NO.	Full length (cm)	Main root length (cm)	Root diameter (cm)	Dry weight (g)	Underground/aboveground dry weight
1	43.13 ± 8.25c	1.56 ± 2.08b	0.38 ± 0.16ab	1.4185 ± 1.12b	0.2762 ± 0.09a
2	43.18 ± 4.39c	1.39 ± 0.67b	0.29 ± 0.05b	1.6424 ± 0.39b	0.3076 ± 0.14a
3	58.50 ± 10.97ab	1.24 ± 0.62b	0.33 ± 0.08ab	2.1436 ± 0.98ab	0.3005 ± 0.11a
4	50.31 ± 8.05bc	0.49 ± 0.15b	0.31 ± 0.06ab	1.6025 ± 0.66b	0.2389 ± 0.10a
5	47.25 ± 2.06bc	0.85 ± 0.17b	0.40 ± 0.02ab	1.7379 ± 0.62b	0.2530 ± 0.06a
6	44.91 ± 5.85c	2.39 ± 2.31ab	0.30 ± 0.03b	1.5392 ± 0.26b	0.2043 ± 0.15a
7	39.94 ± 3.86c	0.91 ± 0.18b	0.35 ± 0.08ab	1.3242 ± 0.40b	0.2972 ± 0.21a
8	59.41 ± 14.60ab	4.03 ± 2.27a	0.31 ± 0.06ab	2.5207 ± 1.24ab	0.3194 ± 0.36a
9	48.49 ± 3.93bc	2.11 ± 1.03ab	0.30 ± 0.09b	1.5622 ± 0.10b	0.3306 ± 0.25a
10	38.19 ± 5.42c	0.69 ± 0.21b	0.30 ± 0.05b	0.9399 ± 0.20b	0.3014 ± 0.22a
11	56.44 ± 8.76b	3.65 ± 2.79ab	0.37 ± 0.05ab	2.0068 ± 0.81ab	0.1785 ± 0.06a
12	49.65 ± 1.31bc	1.67 ± 1.58ab	0.43 ± 0.12a	3.0906 ± 2.12a	0.2371 ± 0.27a
13	56.71 ± 6.68b	2.22 ± 2.23ab	0.40 ± 0.12ab	2.5560 ± 0.96ab	0.1827 ± 0.10a
14	52.13 ± 8.26bc	1.26 ± 0.76b	0.35 ± 0.08ab	1.5097 ± 0.36b	0.2886 ± 0.16a
15	58.85 ± 5.14ab	2.63 ± 3.18ab	0.36 ± 0.10ab	1.4626 ± 0.47b	0.1716 ± 0.05a
16	67.46 ± 10.62a	2.48 ± 1.87ab	0.40 ± 0.10ab	2.4802 ± 0.84ab	0.1945 ± 0.15a

Different letters in the same column indicate significant differences (*p* < 0.05).

**FIGURE 2:**
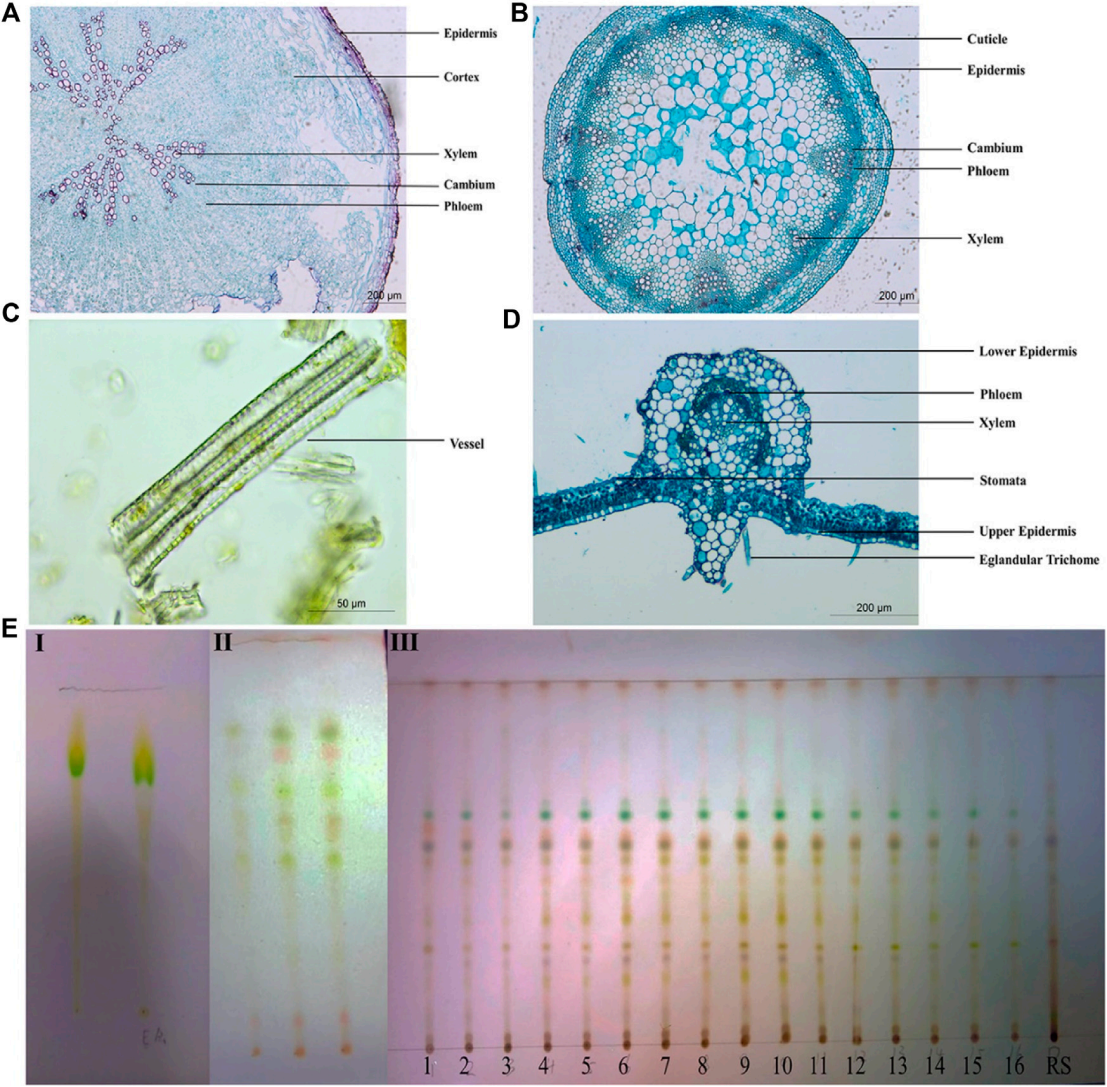
Quality evaluation of *Pimpinella candolleana*. **(A)** Microscopic view of the cross-sectional surface of *Pimpinella candolleana* root. **(B)** Microscopic view of the cross-sectional section of *Pimpinella candolleana* Stem. **(C)** Microscopic view of *Pimpinella candolleana* powder. **(D)** Microscopic view of the cross-sectional section of *Pimpinella candolleana* leaves. **(E)** TLC identification diagram of *Pimpinella candolleana*, I: Chloromethane-methanol (1: 1), II: Ethylacetate-petroleum ether (1: 1), III: Ethylacetate-petroleum ether (0.4:1).

Transverse sections of the root, stem, and leaf of P. candolleana were prepared and subsequently stained with safranin and aniline blue for microscopic analysis. Under a light microscope, the characteristic structures of P. candolleana were identified. In the transverse section of the root, the cork layer exhibited rectangular and brown‒yellow cells. The cortex appeared wide with an irregular arrangement, while the cambium displayed an annular arrangement. The xylem was found to be irregularly arranged radially (Figure 2A). Moving to the transverse section of the stem, the parenchyma cells in the pith region appeared larger, the cortex was narrow, and the fibrous cell wall exhibited lignification. The cambium was notably arranged in a ring-like pattern (Figure 2B). In the transverse section of the leaf, the epidermis was regular in shape, forming rectangles; the xylem was located near the axis, while the phloem was located far from the axis (Figure 2D). In addition, medicinal materials were ground with a mortar and pestle to prepare apricot leaf windbreak powder samples; these powder samples were added with glycerin drops on glass slides and observed under an optical microscope; reticulate vessels, tracheids, calcium oxalate crystals and fiber bundles were found in the powder (Figure 2C).

#### TLC identification and chemical fingerprinting

For the identification of *P. candolleana* medicinal material, thin layer chromatography (TLC) was employed as a widely used technique to determine the chemical fingerprints of plant extracts. A comparison was made between the color, size, and position of spots of *P. candolleana* and its control medicinal materials using different developing agents and color development methods. Initially, a mixture of chloroform and methanol was used as the developing agent, but it was unable to effectively separate the components of *P. candolleana* (Figures 2EI), subsequently, a mixture of ethyl acetate and petroleum ether, a commonly used and less toxic developing agent, was used, showing better results than the chloroform-methanol system (Figures 2EII); based on this, by adjusting the polarity of the developing agent, a mixture of ethyl acetate and petroleum ether (0.4:1) was selected as the developing agent, with 10% sulfuric acid ethanol as the visualization agent (Figures 2EIII). This optimized system enhanced the resolution of *P. candolleana*’s components, rendering the spots more discernible. The congruence observed between the experimental outcomes and the reference standards underscores the effectiveness of TLC in the precise identification of *P. candolleana* medicinal materials.

#### Physicochemical properties of *Pimpinella candolleana*

The moisture content of *P. candolleana* ranged from 5.01% to 6.93%, with an average value of 5.53%. The total ash content varied between 9.28% and 12.55%, with an average value of 10.99%. The water-soluble extractive content ranged from 24.25% to 29.50%, with an average value of 26.72%. The concentration of glucose in the solution ranged from 3.000 to 15.000 μg/mL, and the polysaccharide content ranged from 1.04% to 2.66%, with an average value of 1.57% (Table 2). Notably, *P. candolleana* exhibited a high total ash content, indicating a significant presence of inorganic substances. The high water-soluble extract content aligns with the traditional usage of *P. candolleana* by the Gelao people, who utilized it by soaking it in rice washing water to treat ulcerative colitis. This suggests that the majority of its active ingredients are water soluble. Moreover, the relatively low polysaccharide content in *P. candolleana* is expected since it is used as a whole herb medicine, with the main storage sites for polysaccharides typically found in fruits and roots. These findings suggest that polysaccharides may not be the primary active ingredient in *P. candolleana*.

**TABLE 2:** Content determination results of *Pimpinella candolleana*.

NO.	Content (%)				NO.	Content (%)			
	Total ash	Moisture	Extract	Total polysaccharide		Total ash	Moisture	Extract	Total polysaccharide
1	12.55 ± 0.38a	5.44 ± 0.09bcd	26.29 ± 1.17bc	2.22	9	11.23 ± 0.23b	5.06 ± 0.14cd	27.39 ± 1.51b	1.85
2	10.98 ± 0.25b	5.29 ± 0.05cd	26.63 ± 0.71bc	1.23	10	11.09 ± 0.12b	5.19 ± 0.07cd	26.00 ± 0.85bc	1.42
3	11.07 ± 0.07b	6.93 ± 0.10a	27.55 ± 1.36a	1.56	11	10.97 ± 0.47b	5.22 ± 0.07cd	24.54 ± 0.67c	1.61
4	10.93 ± 0.45b	5.35 ± 0.05cd	27.69 ± 2.29a	1.78	12	11.03 ± 0.08b	5.62 ± 0.04bc	27.48 ± 0.66b	1.04
5	10.03 ± 0.13c	5.62 ± 0.12bc	29.50 ± 0.23a	2.66	13	10.13 ± 0.08c	5.72 ± 0.06b	25.91 ± 0.61bc	1.09
6	11.36 ± 0.17b	5.48 ± 0.46bcd	27.50 ± 1.22a	1.62	14	11.05 ± 0.12b	5.27 ± 0.24d	27.61 ± 0.63b	1.62
7	10.88 ± 0.38b	5.46 ± 0.11bcd	27.02 ± 1.96b	1.63	15	11.16 ± 0.40b	5.10 ± 0.02d	26.12 ± 0.60bc	1.26
8	9.28 ± 0.28d	6.76 ± 0.04a	26.08 ± 1.10bc	1.30	16	12.06 ± 0.36a	5.01 ± 0.17d	24.25 ± 0.69c	1.26

Different letters in the same column indicate significant differences (*p* < 0.05).

#### Identification of the components of *Pimpinella candolleana*

**FIGURE 3:**
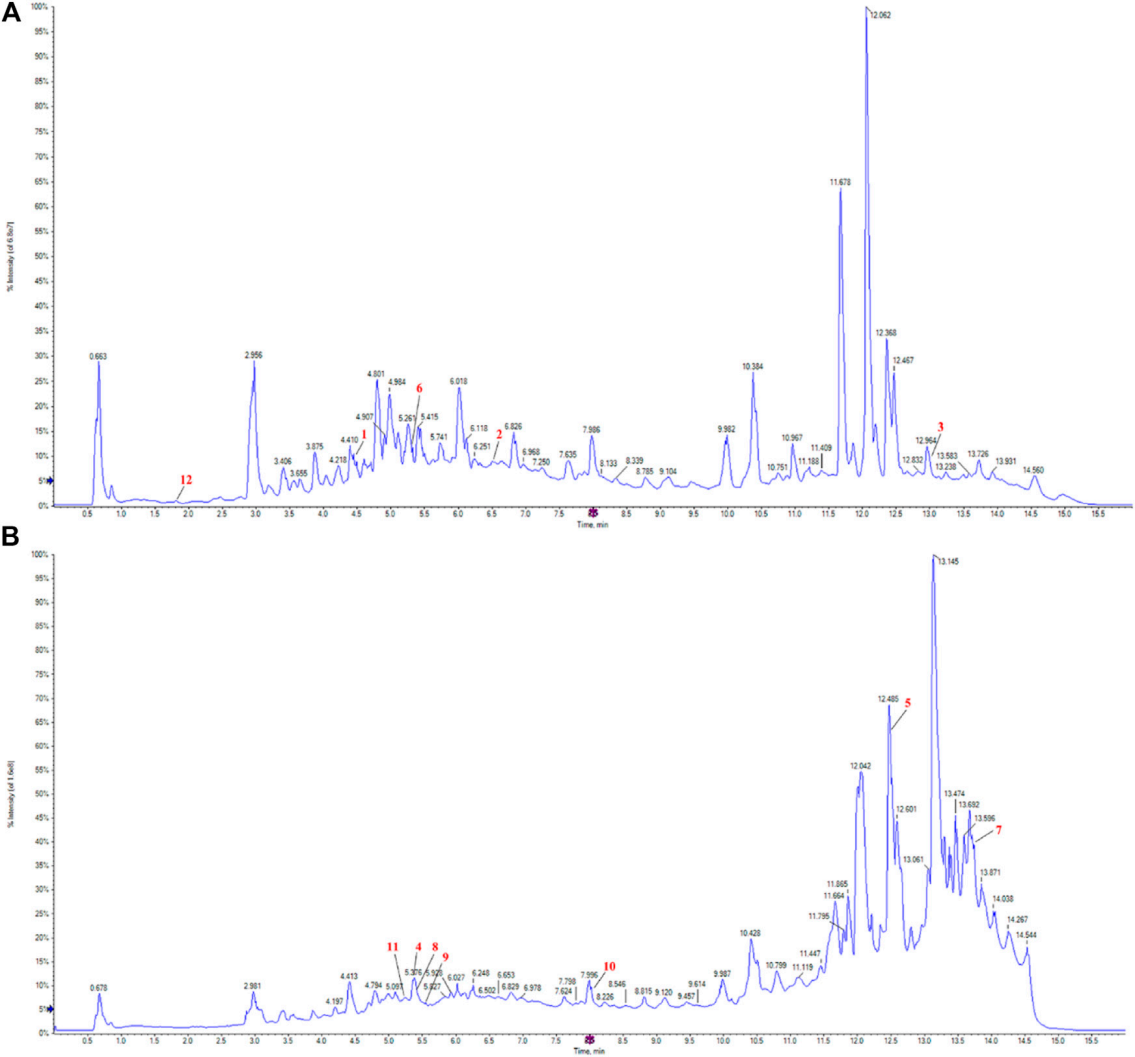
*Pimpinella candolleana* UPLC-Q-TOF-MS total ion flow diagram, **(A)** *Pimpinella candolleana* UPLC-Q-TOF-MS positive ion diagram, **(B)** *Pimpinella candolleana* UPLC-Q-TOF-MS negative ion diagram. The *x*-axis and *y*-axis represent time (min) and intensity, respectively. The red labels marked 1-12 in the figure correspond to the 12 effective chemical components of *Pimpinella candolleana* against ulcerative colitis, as shown in Table 3.

Figures 3A, B present the positive and negative ion spectra, respectively. To determine the structure of metabolites in the samples, molecular weight matching (within a 14 ppm error), secondary fragmentation spectrum analysis, and retention time comparison were performed using the plant metabolomic database. A comprehensive identification of 570 metabolites was achieved from the positive and negative ion spectra of *P. candolleana*. These metabolites primarily include coumarins, organic acids, flavonoids, terpenoids, cinnamic acids, benzoic acids, quinolines, and alkaloids (Supplementary Materials S1, S2).

### Network pharmacology analysis

#### Screening of active components and action targets of *Pimpinella candolleana*

**FIGURE 4:**
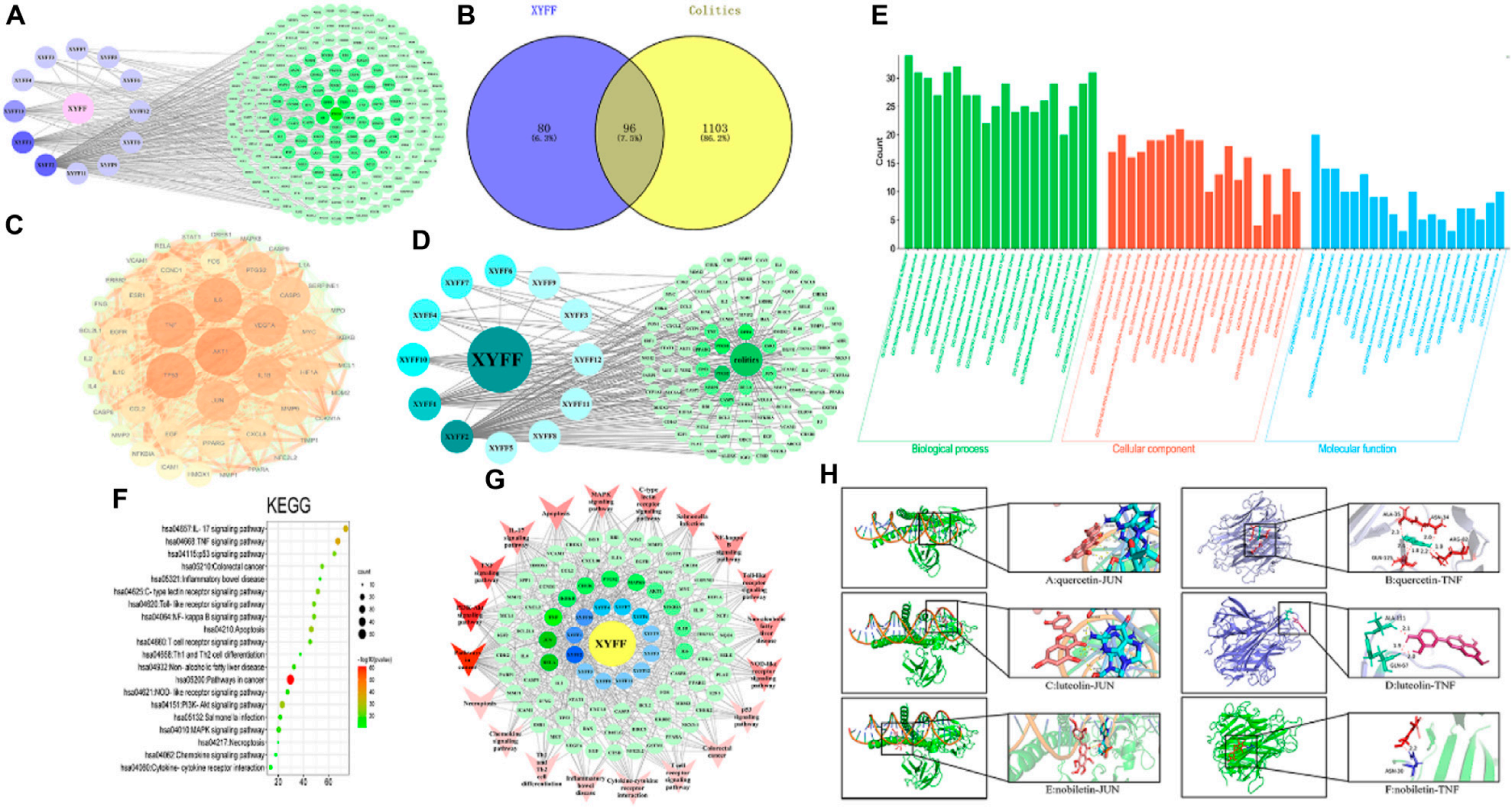
Network pharmacology analysis. **(A)** The active ingredient-target network diagram. The blue on the left represents 12 active ingredients, and the green on the right represents 176 predicted targets of 12 active ingredients. **(B)** Venn diagram of colitis and active ingredients. Blue on the left represents 80 target genes of active ingredients, yellow on the right represents 1103 target genes of colitis, and 96 target genes shared by active ingredients and colitis. **(C)** PPI network diagram based on Cytoscape 3.7.0. **(D)** *Pimpinella candolleana*-active ingredient-target-colitis network. The circle on the left is the ingredient of *Pimpinella candolleana*, and the octagon on the right is the common target of colitis and *Pimpinella candolleana*. The dark color in the middle has a large degree value. **(E)** Histogram of GO enrichment analysis of *Pimpinella candolleana*-uc genes, including the first 20 important enrichment terms of BP, CC and MF, with the abscissa representing the functional information of BP, CC and MF and the ordinate representing the number of genes. **(F)** Bubble chart of the KEGG enrichment pathway with the abscissa representing the proportion of genes of interest in the entries and the ordinate representing each entry. A larger dot size indicates more annotated genes in the entry, and a red dot indicates a lower Q value. **(G)** Target gene-pathway network diagram. The red color at the periphery of the figure represents the related disease pathways, the yellow color at the innermost circle represents *Pimpinella candolleana*, and the blue color is the active component of *Pimpinella candolleana*. The green circle is a common gene, and the degree value with deep node color is large. **(H)** Molecular docking of active components and core targets, docking model of quercetin, luteolin and nobiletin with JUN and TNF-α. Effect model of quercetin, luteolin and nobiletin on JUN: A and B are the effect of quercetin on JUN and TNF-α, respectively; C and D are the effects of luteolin on JUN and TNF-α, respectively. E and F are the relative concentrations of nobiletin to JUN and TNF-α, respectively.

Thirteen components were identified by UPLC-Q-TOF-MS, among which one component did not have any predicted target. The remaining twelve components were assigned numbers and are listed in Table 3. Using the plant metabolomic database, we predicted 176 targets for the 12 components, providing information on plant metabolites and their interactions with human proteins (Figure 4A). To identify potential target genes associated with ulcerative colitis (UC), we obtained 5,492 UC-related genes from the GeneCards database (https://www.genecards.org/) using “Colitis” as the keyword. We then screened for 1,199 significant disease target genes for UC by setting a relevance score threshold of ≥1.1756. The relevance score indicates the strength of association between a gene and a disease based on multiple sources of evidence. By employing a Venn diagram, we visualized the overlap between the 1,199 important target genes for UC and the 176 drug target genes of the *P. candolleana* extract, resulting in the identification of 96 common target genes (Figure 4B).

**TABLE 3:** Content determination results of *Pimpinella candolleana*.

NO.	Mol ID	Molecule name	OB (%)	DL	Molecule structure	Herbs
XYFF1	MOL000006	Luteolin	36.16	0.25	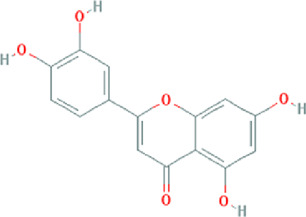	Dictamni Cortex/Herba Patriniae
XYFF2	MOL000098	Quercetin	46.43	0.28	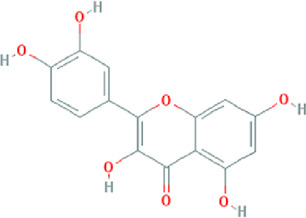	Folium Artemisiae Argyi/Ginkgo Semen
XYFF3	MOL001297	Trans-gondoic acid	30.7	0.2	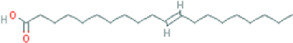	Broussonetiae Fructus/Allii Tuberosi Semen
XYFF4	MOL001944	Marmesin	50.28	0.18	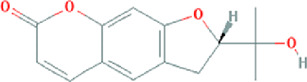	Saposhnikoviae Radix/Peucedani Radix
XYFF5	MOL002032	DNOP	40.59	0.4	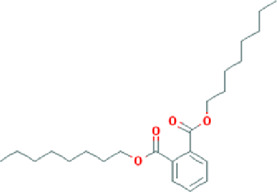	Arecae Semen/Ginsen Radix Et Rhizoma Rubra
XYFF6	MOL002322	isovitexin	31.29	0.72	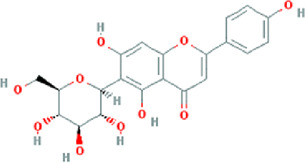	Isatidis Radix/Belamcandae Rhizome
XYFF7	MOL002881	Diosmetin	31.14	0.27	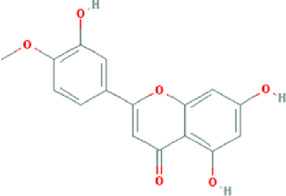	Menthae Herba/Chrysanthemi Flos
XYFF8	MOL004355	Spinasterol	42.98	0.76	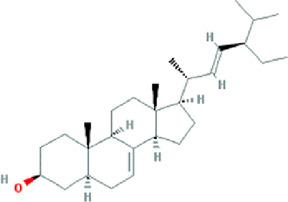	Codonopsis Radix/Gynostemmae Pentaphylli Herba
XYFF9	MOL004792	Nodakenin	57.12	0.69	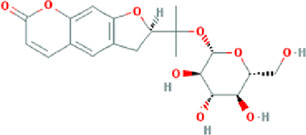	Radix Angelicae Biseratae/Peucedani Radix
XYFF10	MOL005828	Nobiletin	61.67	0.52	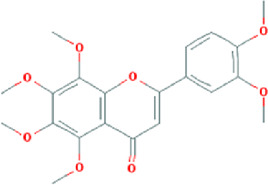	Citrus Reticulata/Aurantii Fructus
XYFF11	MOL006710	8-(beta-D-Glucopyranosyloxy)-7-hydroxy-6-methoxy-2H-1-benzopyran-2-one	36.76	0.42 j	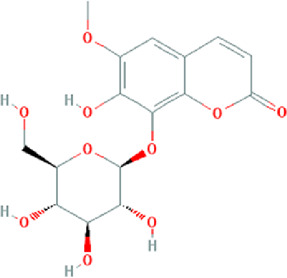	Fraxini Cortex/Semen Aesculi
XYFF12	MOL006967	Beta-D-Ribofuranoside, xanthine-9	44.72	0.21	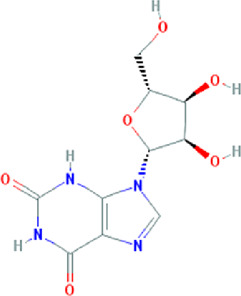	Arum Ternatum Thunb

OB, Oral Bioavailability; DL, Drug-likeness; NO, Number; XYFF, represents *Pimpinella candolleana*.

#### Protein–protein interaction network analysis

To explore the interactions among the common target genes of *P. candolleana* extract and ulcerative colitis (UC), we conducted protein‒protein interaction (PPI) network analysis. The PPI network consisted of 96 nodes and 2,031 edges, with an average degree of 42.3 and an average clustering coefficient of 0.76. The *p*-value, which was found to be less than 1.0e-16, indicated strong interactions among the common target genes of the *P. candolleana* extract and UC. Figure 4C illustrates the PPI network diagram, where each node represents a gene, and each edge represents an interaction between two genes. The nodes with larger sizes and deeper colors indicate higher degree values, highlighting their importance in the network. Notably, AKT1, TP53, TNF-α, IL6, VEGFA, IL-1β, and JUN exhibited the highest degree values, suggesting their potential vital roles in the mechanisms of *P. candolleana* extract in treating UC.

Furthermore, Figure 4D presents the drug-active component-target gene-UC network diagram. By identifying the relevant nodes of the active components, we found that XYFF2 (quercetin), XYFF1 (luteolin), and XYFF10 (nobiletin) were associated with 81, 37, and 17 UC target genes, respectively. This indicates that these components may have significant effects on UC treatment.

#### GO and KEGG pathway enrichment analyses

To gain comprehensive insights into the biological functions and pathways associated with the common target genes of *P. candolleana* extract and ulcerative colitis (UC), we performed gene ontology (GO) and Kyoto Encyclopedia of Genes and Genomes (KEGG) pathway enrichment analyses. The results revealed that the common target genes play crucial roles in various biological processes, including responses to inorganic substances, xenobiotic stimuli, radiation, lipopolysaccharide, and infection. They are also involved in diverse cellular components, such as the transcription regulator complex, membrane raft, membrane microdomain, RNA polymerase II transcription regulator complex, and vesicle lumen. Additionally, the molecular functions of these target genes encompass cytokine receptor binding, DNA-binding transcription factor binding, cytokine activity, RNA polymerase II-specific DNA-binding transcription factor activity, and receptor ligand activity (Figure 4E).

Moreover, the KEGG pathway analysis highlighted the involvement of the common target genes in a wide range of biological pathways, including pathways in cancer (hsa05200), TNF-α signaling pathway (hsa04668), PI3K-Akt signaling pathway (hsa04151), apoptosis (hsa04210), p53 signaling pathway (hsa04115), IL-17 signaling pathway (hsa04657), and C-type lectin receptor signaling pathway (hsa04625), among others (Figure 4F). Notably, 20 pathways exhibited a log10 *p*-value of ≤ −10, indicating the significant participation of the common target genes in these pathways.

Furthermore, through the analysis of the target-pathway network, we observed that XYFF2 (quercetin), XYFF1 (luteolin), and XYFF10 (nobiletin) were associated with 81, 37, and 17 UC target genes, respectively, suggesting their potential significant impact on UC treatment. Additionally, we identified that *P. candolleana* extract mainly acts on RELA, JUN, TNF-α, IKBKB, CHUK, PTGS2, and MAPK8 through the active components XYFF2 (quercetin), XYFF1 (luteolin), and XYFF10 (nobiletin) (Figure 4G). These findings provide valuable insights into the potential mechanisms underlying the anti-inflammatory and therapeutic effects of *P. candolleana* extract on UC. The identification of active components, their associated target genes, and regulatory pathways enhances our understanding of the pharmacological activities of *P. candolleana* extract and lays the groundwork for the development of novel therapeutic strategies for UC.

#### Molecular docking verification

Three active components with the highest degree values (quercetin, luteolin, and nobiletin) and the six target proteins with the highest degree values (RELA, JUN, TNF-α, IKBKB, CHUK, and PTGS2) were selected for the analysis. The results revealed favorable binding, as all three active components exhibited negative interaction energies with the target proteins, indicating strong affinity (Table 4). Notably, quercetin demonstrated particularly potent binding affinity with JUN and TNF-α, with interaction energies lower than −5 kcal/mol (−5.33 and −5.32, respectively), suggesting its potential therapeutic efficacy (Figure 4H). These findings provide further support for the anti-inflammatory and therapeutic effects of *P. candolleana* extract on UC and indicate the potential of the identified active components and target proteins as valuable therapeutic targets for UC treatment.

**TABLE 4:** Docking binding energy between surface active ingredients and core target molecules.

Ingredients	CAS	Target	PDB ID	Binding energy (kcal/mol)
Quercetin	117-39-5	RELA	1NFI	−3.77
		PTGS2	5IKV	−2.85
		TNF-α	1A8 M	−5.32
		IKBKB	3BRT	−2.42
		CHUK	5TQW	−2.89
		JUN	1A02	−5.33
Luteolin	491-70-3	RELA	1NFI	−4.03
		PTGS2	5IKV	−3.47
		TNF-α	1A8 M	−4.66
		IKBKB	3BRT	−2.98
		CHUK	5TQW	−3.26
		JUN	1A02	−4.96
Nobiletin	478-01-3	RELA	1NFI	−3.45
		PTGS2	5IKV	−2.20
		TNF-α	1A8 M	−4.18
		IKBKB	3BRT	−2.72
		CHUK	5TQW	−2.31
		JUN	1A02	−4.20

### *Pimpinella candolleana* Anti-UC experiment

#### Effects of *Pimpinella candolleana* on body weight and DAI score of UC rats

The effects of *P. candolleana* on body weight and disease activity index (DAI) score were evaluated in UC rats induced by acetic acid. Following acetic acid induction, rats in the normal group displayed normal water and food intake, feces, back hair, and mental condition, with a tendency for increased body weight. In contrast, rats in the other groups exhibited significantly reduced water and food intake, curled-up bodies, and gray, lusterless fur. Their stools were shapeless or bloody, and they displayed signs of loose or bloody stools around the anus. The body weights of the model group and *P. candolleana* groups continued to decrease before *P. candolleana* administration, but 3 days after administration, the body weights of rats in all *P. candolleana* groups began to gradually increase. On the day prior to administration, the DAI scores of all groups were significantly higher than those of the normal group (*p* < 0.01). However, by the ninth day of *P. candolleana* treatment, the high-dose group exhibited a significantly lower DAI score than the model group (*p* < 0.01). By the 13th day, the DAI score of the high-dose *P. candolleana* group tended to reach 0, while the DAI scores of the other *P. candolleana* groups were significantly lower than that of the model group (*p* < 0.01) and tended to normalize. These findings indicate that *P. candolleana* intervention effectively alleviates the symptoms of acetic acid-induced UC in rats, as evidenced by improvements in body weight and DAI score (Figure 6A).

#### Influence on organ index and pathology

**FIGURE 5:**
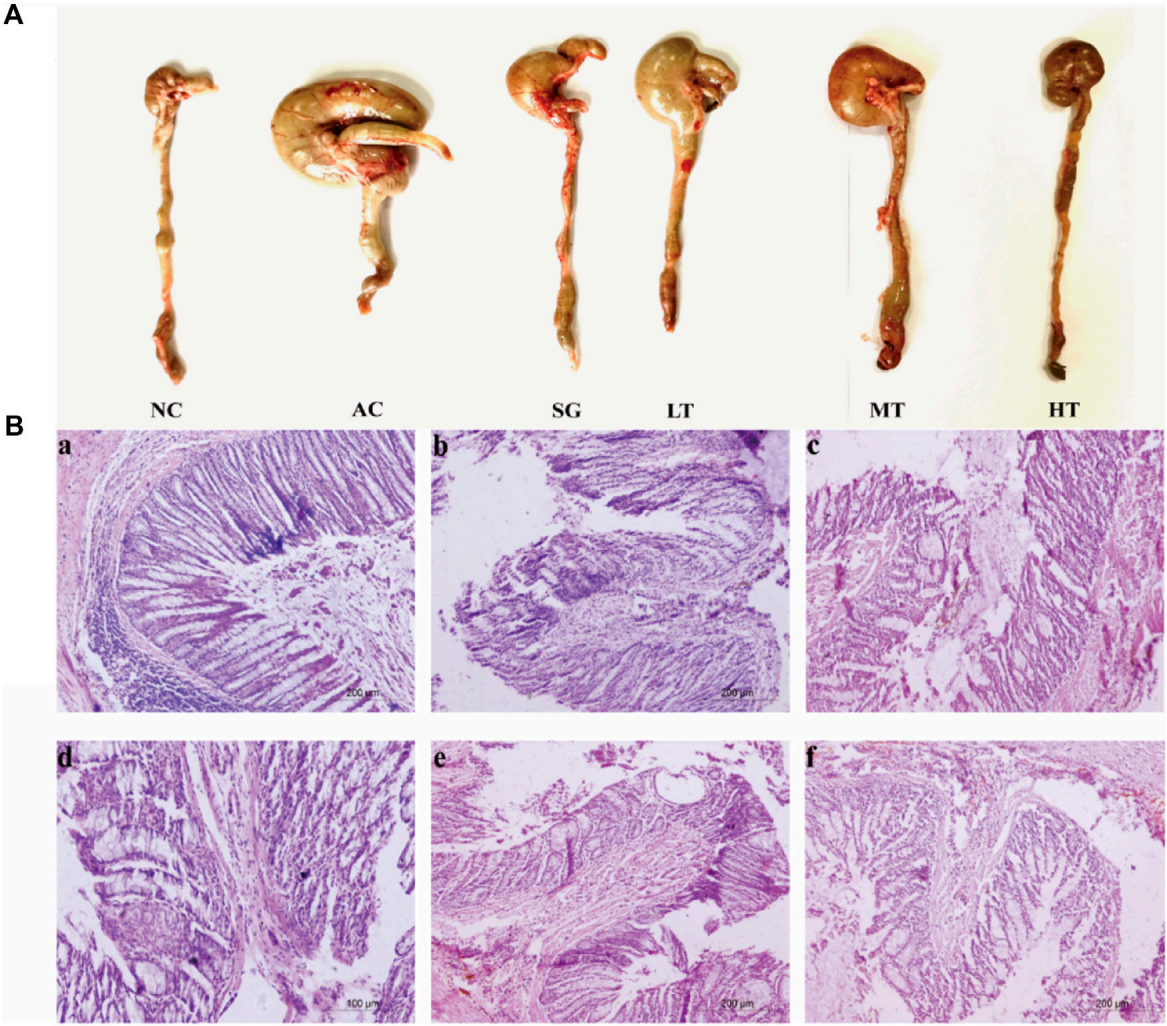
Effect of *Pimpinella candolleana* on UC. **(A)** Comparison of the colon before and after medication (AC: Model group. SG: Sulfasalazine group. LT: *Pimpinella candolleana* low-dose group. MT: *Pimpinella candolleana* mid-dose group. HT: *Pimpinella candolleana* high-dose group). **(B)** HE staining of the colon **(a)**: Normal group. **(b)**: Model group. **(c)**: Sulfasalazine group. **(d–f)** represent the *Pimpinella candolleana* low-, medium- and high-dose groups, respectively).

**FIGURE 6:**
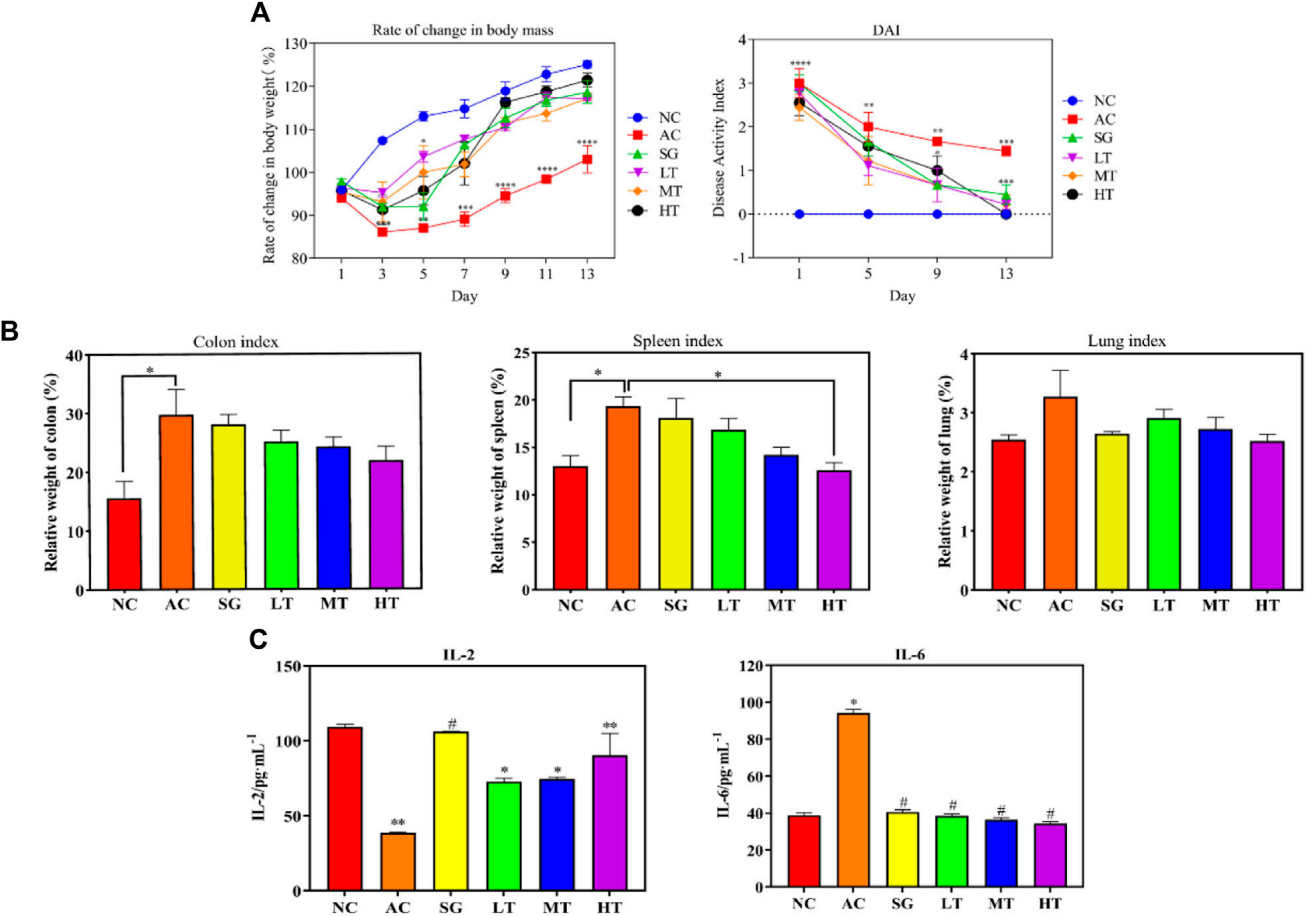
*Pimpinella candolleana* Anti-UC Experiment. **(A)** Influence of body weight and DAI, *Pimpinella candolleana*’s effect on the body weight of UC rats (Abscissa: time, Ordinate: Rate of change in weight), *Pimpinella candolleana*’s effect on the DAI of rats with UC (Abscissa: treatment time, Ordinate: DAI. **(B)** Effect of *Pimpinella candolleana* on the organ index of UC rats. **(C)** Effect of *Pimpinella candolleana* on IL-2 and IL-6 in ulcerative colitis. (AC: Model group. SG: Sulfasalazine group. LT: *Pimpinella candolleana* low-dose group. MT: *Pimpinella candolleana* mid-dose group. HT: *Pimpinella candolleana* high-dose group). (Compared to the NC group, *p < 0.05, **p < 0.01, ***p < 0.001, ****p < 0.0001; Compared to the AC group, #*p* < 0.05, ##*p* < 0.01, ###*p* < 0.001, ####*p* < 0.001).

To evaluate the impact of ulcerative colitis (UC) and the potential protective effects of *P. candolleana*, organ indices were measured in different rat groups. The colon index, spleen index, and liver index were significantly increased in all acetic acid-induced rats compared to the normal group. However, treatment with *P. candolleana* led to a dose-dependent reduction in these indices (*p* < 0.05), indicating its protective effects against UC-induced organ damage. Remarkably, the high-dose group of *P. candolleana* exhibited the most pronounced decrease in these indices (Figure 6B). Histopathological examination of colon tissue using hematoxylin and eosin staining revealed severe damage to the colon epithelium and extensive inflammatory infiltration in the model group, in contrast to the normal group. Notably, treatment with *P. candolleana* ameliorated colonic injury and reduced inflammatory infiltration in UC rats, indicating its potential to mitigate histopathological damage caused by ulcerative colitis (Figures 5A, B).

#### Effects of *Pimpinella candolleana* on serum cytokine levels in UC rats

To elucidate the anti-inflammatory mechanism of *P. candolleana*, we evaluated the serum levels of IL-2 and IL-6, two key cytokines involved in UC pathogenesis. Compared with the normal group, the model group rats had significantly lower serum levels of IL-2 and markedly higher levels of IL-6, reflecting an inflammatory response induced by acetic acid in UC rats. *Pimpinella candolleana* treatment restored the serum levels of IL-2 and IL-6 in a dose-dependent manner. The high-dose group of *P. candolleana* had a normalized serum level of IL-6. These results demonstrated a clear dose-dependent effect of *P. candolleana* on modulating the serum levels of inflammatory cytokines (Figure 6C).

#### Effects of *Pimpinella candolleana* on the mRNA expression of key genes involved in UC rats

**FIGURE 7:**
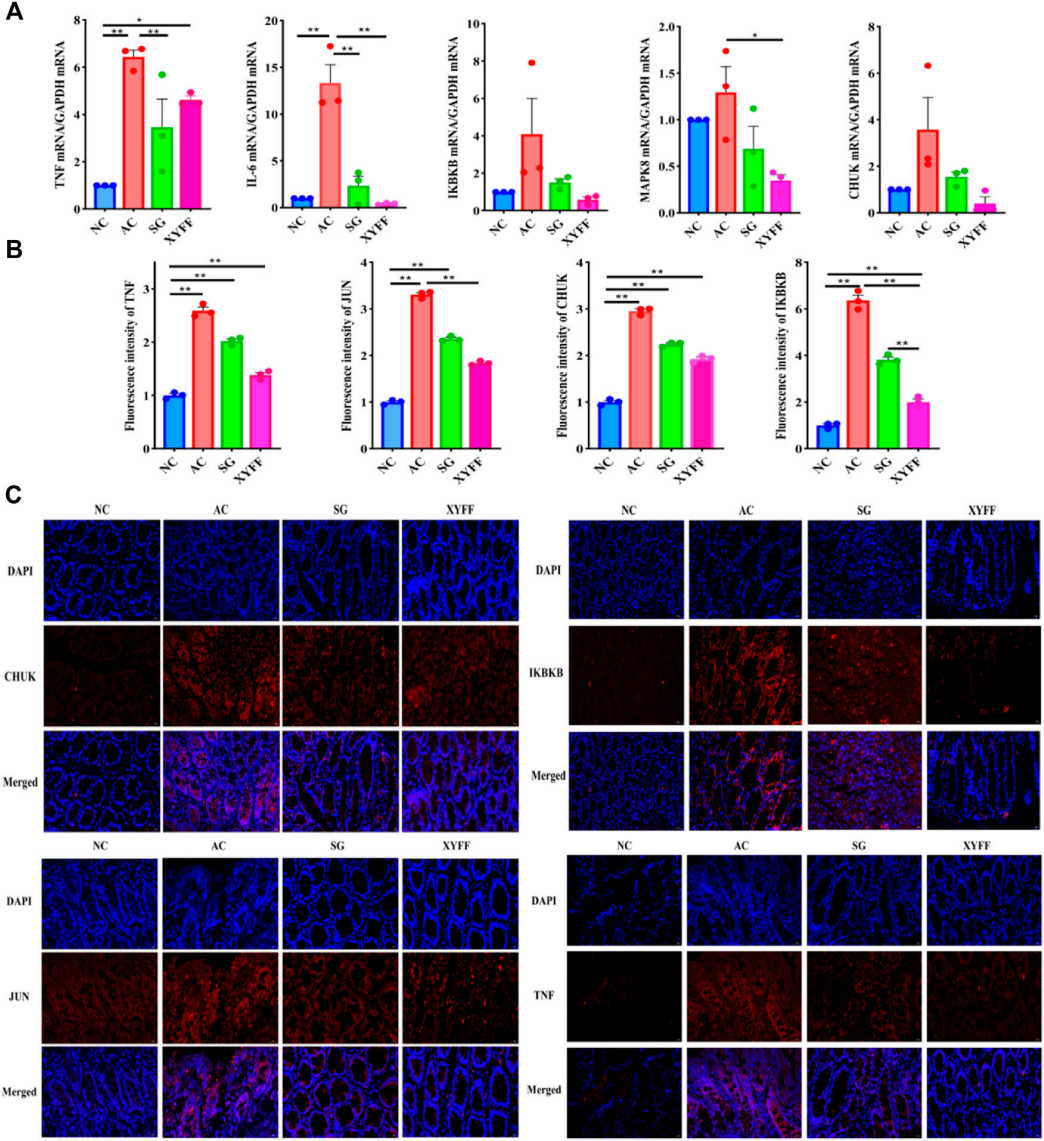
*Pimpinella candolleana* alleviates inflammation in the colon tissue of UC rats. **(A)** Expression levels of CHUK, IKBKB, JUN, TNF-α, and IL-6 in colon tissue were detected by RT-QPCR. **(B)** Fluorescence intensity values of CHUK, IKBKB, JUN, and TNF-α expression in colon tissue were measured by immunofluorescence staining. **(C)** Expression levels of CHUK, IKBKB, JUN, and TNF-α in colon tissue were detected by immunofluorescence staining. Glyceraldehyde-3-phosphate dehydrogenase (GAPDH) served as an internal control. AC: Model group, SG: Sulfasalazine group, XYFF: *Pimpinella candolleana* group. Data are presented as the mean ± SD. *p < 0.05, **p < 0.01 compared with the control group. (Original magnification, ×40, bar = 100 μm). (*p < 0.05, **p < 0.01).

The mRNA expression of key genes (IL-6, MAPK8, TNF-α, CHUK, and IKBKB) was measured to investigate the molecular mechanisms underlying the anti-inflammatory action. The mRNA expression of these genes was significantly increased in the colon tissue of the model group rats compared to the normal group rats, indicating that they were upregulated by acetic acid-induced inflammation. However, *P. candolleana* treatment decreased the mRNA expression of these genes in a dose-dependent manner, suggesting that *P. candolleana* could regulate the expression of these genes and counteract their proinflammatory effects. These results demonstrate that *P. candolleana* can suppress the mRNA expression of the proinflammatory cytokines IL-6, MAPK8, TNF-α, CHUK, and IKBKB in the colon tissue of UC rats and attenuate inflammation in colon tissue (Figure 7A). These findings are consistent with our previous results and provide further support for the anti-inflammatory properties of *P. candolleana*. Specifically, our results suggest that *P. candolleana* can downregulate the expression of key proinflammatory genes and reduce inflammation in the colon tissue of UC rats. These results contribute to our understanding of the underlying molecular mechanisms of *P. candolleana*’s therapeutic effects on UC and suggest that it may serve as a promising therapeutic agent for the treatment of UC.

#### Immunofluorescence detection of TNF-α, IKBKB, JUN, and CHUK expression in colon tissue

To confirm the effects of *P. candolleana* on the protein expression of the key genes involved in UC pathogenesis, we performed immunofluorescence staining of TNF-α, IKBKB, JUN, and CHUK in the colon tissue of rats. The immunofluorescence images (Figures 7B, C) showed that the fluorescence intensity of TNF-α, IKBKB, JUN, and CHUK was significantly higher in the colon tissue of the AC group rats than in the NC group rats (*p* < 0.01), indicating that they were overexpressed by acetic acid-induced inflammation. However, *P. candolleana* treatment decreased the fluorescence intensity of these proteins in a dose-dependent manner, indicating that *P. candolleana* could suppress their overexpression and inhibit their proinflammatory effects.

## Discussion

Currently, western drugs commonly used to treat UC have certain limitations, highlighting an urgent need to develop new anti-UC drugs. *Pimpinella candolleana*, a traditional herbal medicine used by the Gelao ethnic group, has shown efficacy in treating UC. However, few studies have evaluated the quality and explored the anti-UC mechanism of *P. candolleana*. Therefore, this study aims to investigate the anti-UC mechanism of *P. candolleana* through quality evaluation, network pharmacology, and data mining combined with *in vivo* experiments.

*Pimpinella candolleana* can be easily confused with other medicinal plants due to similarities in identification characteristics, such as Seseli mairei Wolff, resulting in adulteration and affecting its efficacy (Gui et al., 1991). Comprehensive pharmacognostic studies of *P. candolleana* are scarce, motivating quality evaluation to support conservation and utilization of this valuable ethnic Chinese medicinal material. We observed the morphological features, dry weights, and underground-to-aboveground dry weight ratios of *P. candolleana* plants collected from Guizhou Province, China. The results showed slight differences in characteristics potentially related to environmental factors influencing plant growth and development (Wei and Gui, 1992; Kuang et al., 2023). Therefore, it is necessary to standardize the cultivation, harvesting methods and harvesting time of *P. candolleana* to ensure the consistency of its quality. We also prepared cross-sections and powder samples of *P. candolleana* and identified its characteristic structures by optical microscopy, such as reticulate vessels, rectangular wood parenchyma cells and rectangular cork cells. These structures can be used as microscopic identification characteristics of *P. candolleana* medicinal material (Su, 2000). In TLC identification, some developing agents used in the Chinese Pharmacopoeia have high toxicity and poor separation degree for *P. candolleana* medicinal material. By changing the solvent polarity, we used a mixed system of ethyl acetate and petroleum ether with low toxicity and wide application as the developing system and 10% ethanol sulfuric acid as the developer. We established a TLC chromatogram of *P. candolleana* with the best separation effect and clear spots, which can also be used to identify the authenticity of *P. candolleana* medicinal material. In addition, we determined the moisture content, total ash content, water-soluble extractive content and polysaccharide content of *P. candolleana* medicinal material. We found that *P. candolleana* had high contents of inorganic substances and water-soluble extractives but a low content of polysaccharides, indicating that most of the active ingredients of *P. candolleana* were water soluble, which may be an important basis for folk people often using decoctions to treat UC (He et al., 2023). Based on these results, we established a preliminary quality evaluation standard for *P. candolleana* medicinal material that includes macroscopic identification (morphological features), microscopic identification (characteristic structures), physicochemical identification (moisture content, total ash content, water-soluble extractives content and polysaccharide content) and TLC identification (chemical fingerprints). This standard can provide a reference for the quality control and standardization of *P. candolleana* medicinal material.

We employed an acetic acid-induced UC rat model to demonstrate the therapeutic effect of *P. candolleana* extract via animal experiments. We found that *P. candolleana* extract could effectively alleviate the symptoms of acetic acid-induced UC in rats, as evidenced by the improvement in body weight and DAI score (Cho et al., 2011; Ren et al., 2023), indicating that it could improve their nutritional status and clinical symptoms. The high-dose group of *P. candolleana* extract showed the most significant reduction in these indices among all treatment groups. The colon index reflects the degree of colon inflammation and edema (Lv et al., 2015). The spleen index reflects the immune response to inflammation (Ji et al., 2020). The liver index reflects liver function and metabolism (Zhu et al., 2019). Our results suggest that *P. candolleana* extract could ameliorate inflammation and edema in the colon, modulate the immune response of the spleen, and improve liver function and metabolism in UC rats. Histopathology is the gold standard for diagnosing UC and evaluating its severity (Ae et al., 2023). The histological features of UC include mucosal erosion, ulceration, crypt abscesses, goblet cell depletion, and inflammatory cell infiltration (Bressenot et al., 2015). Our results demonstrate that *P. candolleana* extract could improve mucosal integrity and reduce inflammatory cell infiltration in the colon tissue of UC rats.

UPLC-Q-TOF-MS qualitatively analyzed 570 metabolites in *P. candolleana* extract, mainly including coumarins, organic acids, flavonoids, terpenoids, cinnamic acids, benzoic acids, quinolines and alkaloids (Zhao et al., 2007). These metabolites are consistent with previous reports on the chemical constituents of *P. candolleana* (Wei et al., 2005; Li et al., 2023). Among these metabolites, some have been reported to have anti-inflammatory and antioxidant effects, such as flavonoids (quercetin, luteolin, and nobiletin), coumarins (esculetin and scopoletin) and terpenoids (ursolic acid) (Wang et al., 2020; Rong et al., 2021; Chiang et al., 2022). These metabolites may be responsible for the pharmacological activities of *P. candolleana*.

To elucidate the anti-inflammatory mechanism of *P. candolleana* extract, we evaluated the serum levels of IL-2 and IL-6, both of which are implicated in UC pathogenesis. IL-2 and IL-6 levels were significantly decreased in the model group compared with the normal group, reflecting an inflammatory response induced by acetic acid in UC rats. *Pimpinella candolleana* extract treatment restored the serum levels of IL-2 and IL-6 in a dose-dependent manner. The high-dose *P. candolleana* extract group had a normalized serum level of IL-6. These results demonstrated a clear dose-dependent effect of *P. candolleana* extract on modulating the serum levels of inflammatory cytokines. IL-2 and IL-6 play important roles in the pathogenesis of UC (Owusu et al., 2020; Cuellar-Núñez et al., 2021). IL-2 is involved in the activation and differentiation of T cells and B cells and regulates the balance between Th1 and Th2 cells (Barron et al., 2010). IL-6 is involved in the activation of macrophages and neutrophils and stimulates the production of acute phase proteins (Jin et al., 2013; Seledtsov et al., 2019). Our research results show that the extract of *P. candolleana* can promote IL-2 and inhibit the production of IL-6 in UC rats and reduce inflammation.

To further identify the key components and targets of *P. candolleana* in ulcerative colitis and reveal its potential anti-inflammatory pathways and mechanisms, we used network pharmacology to construct a drug-active component-target-UC network and performed GO and KEGG pathway enrichment analysis and molecular docking validation. Through network pharmacology analysis, we collected 96 potential targets of *P. candolleana* for treating UC and found that *P. candolleana* might intervene in UC by regulating inflammatory factor-mediated signaling pathways, protein binding, etc. Subsequently, we used molecular docking to investigate the binding ability of key active components (quercetin, luteolin, and nobiletin) with core target proteins (RELA, JUN, TNF-α, IKBKB, CHUK, PTSG2) and found that the receptors and ligands had low binding energies, providing a strong basis for further exploring the *in vivo* mechanisms. The results of animal RT‒qPCR and immunofluorescence experiments were consistent with the predictions of network pharmacology and molecular docking, confirming our hypothesis and revealing the mechanism of action of *P. candolleana* on UC; that is, *P. candolleana* can treat UC by targeting multiple genes and pathways involved in inflammation. The IKBKB gene-encoded IKK-ß and CHUK gene-encoded IKK-α (Olivotto et al., 2013) are the main kinases that induce NF-κB activation by proinflammatory stimuli. NF-κB is the main regulator of inflammatory gene expression (Adelaja and Hoffmann, 2019) and plays a vital role in a wide range of processes, such as immunity, inflammation, cell development, growth and survival (Caamaño and Hunter, 2002). Tumor necrosis factor α (TNF-α) is a key proinflammatory cytokine that plays an indispensable role in the pathogenesis of UC carcinogenesis. Studies have shown that icariin can alleviate DSS-induced colitis by inhibiting NF-κB signaling pathway-mediated TNF-α and IL-6 expression (Zhang et al., 2021). In conclusion, this study showed that *P. candolleana* extract can treat acetic acid-induced rat UC, reduce colon damage, and regulate the inflammatory response, possibly related to the NF-κB signaling pathway.

## Conclusion

This study used UPLC-Q-TOF-MS in combination with network pharmacology, molecular docking, and experimental verification methods to establish a quality evaluation standard for *P. candolleana* for the first time. The study identified the chemical components of *P. candolleana*, elucidated the interactions among its active components, potential targets, and signaling pathways, and further validated the findings by animal experiments. The results indicated that *P. candolleana* could exert anti-inflammatory effects through multiple components, targets, and pathways. We established an acetic acid-induced colitis rat model to assess the anti-inflammatory properties of *P. candolleana* in rats. *Pimpinella candolleana* treatment significantly improved the body weight and pathological damage to the colon in rats and effectively reduced the DAI score and levels of inflammatory factors. Moreover, *P. candolleana* decreased the expression of IKBKB, JUN, CHUK, and TNF-α proteins, thus exerting anti-inflammatory and therapeutic effects on colitis. The above results have enriched our understanding of the chemical constituents and anti-inflammatory mechanisms of *P. candolleana*. This study provides a powerful tool for the overall quality control of *P. candolleana* and offers objective scientific evidence for the clinical application of *P. candolleana* in the treatment of inflammation-related diseases.

## Data Availability

The raw data supporting the conclusion of this article will be made available by the authors, without undue reservation.

## Abbreviations

BP, Biological process; CC, Cellular component; P. candolleana, Pimpinella candolleana Wight et Arn; UC, Ulcerative colitis; TLC, Thin layer chromatography; DAI, Disease activity index; UPLC-Q-TOF-MS, Ultra-performance liquid chromatography-tandem time-of-flight mass spectrometry; TCM, Traditional Chinese medicine; TCMSP, Traditional Chinese Medicine Systems Pharmacology database and analysis platform; PPI, Protein‒protein interaction network; MF, Molecular function; GO, Gene ontology; KEEG, Kyoto encyclopedia of genes and genomes; IL, Interleukin; TNF-α, Tumor necrosis factor-α; KEGG, Kyoto genome encyclopedia; HE, Histopathological examination; GAPDH, Glyceraldehyde 3-phosphate dehydrogenase; IBD, Inflammatory bowel disease.
